# Mössbauer and EPR detection of iron trafficking kinetics and possibly labile iron pools in whole *Saccharomyces cerevisiae* cells

**DOI:** 10.1016/j.jbc.2024.107711

**Published:** 2024-08-22

**Authors:** Grant Delanoy, Cody Lupardus, Shaik Waseem Vali, Joshua D. Wofford, Shantanu Thorat, Paul A. Lindahl

**Affiliations:** 1 Department of Chemistry, Texas A&M University, College Station, Texas, USA; 2 Department of Biochemistry and Biophysics, Texas A&M University, College Station, Texas, USA; 3 Department of Computer Science and Engineering, Texas A&M University, College Station, Texas, USA

**Keywords:** iron trafficking, Mössbauer spectroscopy, electron paramagnetic resonance, EPR, mathematical kinetic modeling, ICP-MS

## Abstract

The kinetics of iron trafficking in whole respiring *Saccharomyces cerevisiae* cells were investigated using Mössbauer and EPR spectroscopies. The Mössbauer-active isotope ^57^Fe was added to cells growing under iron-limited conditions; cells were analyzed at different times post iron addition. Spectroscopic changes suggested that the added ^57^Fe initially entered the labile iron pool, and then distributed to vacuoles and mitochondria. The first spectroscopic feature observed, ∼ 3 min after adding ^57^Fe plus a 5 to 15 min processing dead time, was a quadrupole doublet typical of nonheme high-spin Fe^II^. This feature likely arose from labile Fe^II^ pools in the cell. At later times (15–150 min), magnetic features due to S = 5/2 Fe^III^ developed; these likely arose from Fe^III^ in vacuoles. Corresponding EPR spectra were dominated by a g = 4.3 signal from the S = 5/2 Fe^III^ ions that increased in intensity over time. Developing at a similar rate was a quadrupole doublet typical of S = 0 [Fe_4_S_4_]^2+^ clusters and low-spin Fe^II^ hemes; such centers are mainly in mitochondria, cytosol, and nuclei. Development of these features was simulated using a published mathematical model, and simulations compared qualitatively well with observations. In the five sets of experiments presented, all spectroscopic features developed within the doubling time of the cells, implying that the detected iron trafficking species are physiologically relevant. These spectroscopy-based experiments allow the endogenous labile iron pool within growing cells to be detected without damaging or altering the pool, as definitely occurs using chelator-probe detection and possibly occurs using chromatographic separations.

Iron has exceptional catalytic properties that render it essential for all eukaryotic organisms, including humans (1, 2, 3, 4). This d-block transition metal serves diverse cellular roles involving mitochondrial respiration, metabolic catalysis, membrane synthesis, and DNA replication/repair. Ironically, these same exceptional properties also damage cells. For example, some Fe^II^ complexes, including aqueous Fe^II^ ions, catalyze side reactions involving Fenton chemistry that generate reactive oxygen species which can damage macromolecules such as DNA, membranes, and proteins (5).

Fe^II^ complexes that promote these nefarious reactions may be members of *Labile Iron Pools* (LFePs) in cells (6). The LFeP in the cytosol, informally called the *Grand Central Station* of iron trafficking, receives nutrient iron from the environment and then delivers it to various client proteins throughout the cell. The LFeP in mitochondria likely serves as feedstock for heme and iron-sulfur-cluster (ISC) assembly in that organelle (7, 8). Vacuoles also contain a LFeP whose redox state (Fe^I^^I^^I^ or Fe^I^^I^) is controlled by whether cells are grown aerobically or hypoxically (9, 10).

Intracellular Fe trafficking is tightly regulated to minimize these deleterious reactions while optimizing the efficiency of protein metallation. Balancing these two opposing objectives requires that such pools be *labile*—*i.e.* composed of iron complexes with coordinating ligands that tend to dissociate to a limited extent. Limited lability is essential for member iron complexes to remain intact during transit but then release their cargo (*i.e.* iron) upon reaching client apo-protein destinations.

How cells do this would be better understood if the chemical composition of these pools were known. The existence of LFePs in eukaryotic cells has been recognized for > 40 years (6, 11, 12), and it’s clear that they are composed of low-molecular-mass (LMM) nonproteinaceous Fe^II^ complexes. However, due to their lability, the chemical composition and exact cellular functions of these pools remain unestablished.

The most popular approach to investigating LFePs involves incubating intact cells with custom-designed fluorescence-based chelator probes (12, 13). When such probes enter cells and bind pool iron, their fluorescence properties change which allows sensitive detection of such pools. The ability of chelator probes to be imported directly into whole metabolically active cells is important, as it allows the LFeP to be investigated without disrupting the cell. On the other hand, these probes destroy the complexes of interest as an inherent part of the detection process (13). Chelator probes are also useful in estimating the *concentration* of LFePs in cells, but those estimates can vary depending on the probe and reaction conditions.

A second popular approach to investigating LFePs involves isolating pool complexes chromatographically (13). Using this approach, we recently reported that the LFeP in *Escherichia coli* appears dominated by Fe^II^ ions coordinated to nucleotide triphosphates and (separately) to citrate (14). Neither aqueous (*i.e.* hexaqua) Fe^II^ nor Fe^II^-glutathione complexes were detected. The latter species is popularly considered to be the dominant or even the exclusive member of the cytosolic LFeP (15, 16, 17).

The chromatography-based approach to characterizing LFePs has the potential of isolating and identifying endogenous LFeP complexes, a clear advantage. However, it also requires that cells be lysed, a disadvantage. Lysates are typically filtered, and flow-through solutions are passed down chromatography columns interfaced to an inductively-coupled-plasma mass spectrometer (LC-ICP-MS). Fractions containing low-molecular-mass Fe complexes can be detected and, in principle, characterized by established downstream methods such as ESI-MS. However, the possibility that these processing steps alter LFeP complexes to generate artifacts cannot be excluded despite all efforts to avoid this. In fact, we recently showed that gently lysing *E. coli* cells alters their LFeP (14). Using Mössbauer (MB) spectroscopy, we found that simply thawing frozen cells (a strain of *E. coli* containing the hemolysin gene) in an MB cup anaerobically and refreezing them 30 min later, without removing the sample from the cup, causes a significant percentage of cellular iron-sulfur clusters (ISCs) to degrade into nonheme high-spin Fe^II^ species (14). Such species likely contribute to the LFeP.

Here we used a third approach to examine the LFeP, involving spectroscopic detection of species within whole intact cells. A similar approach was introduced by Hoffman and Culotta who detected and characterized the pool of nonproteinaceous low-mass Mn^II^ species in yeast cells using electron-nuclear double resonance (ENDOR) spectroscopy (18). We additionally used spectroscopy to examine the kinetics of iron import and trafficking in yeast cells. Although spectroscopic interrogations of metal pools do not allow for their unambiguous chemical identification, they do allow pools to be investigated without concern of generating artifacts.

The combined use of MB and EPR spectroscopies can be used to investigate the oxidation and spin states of iron in complex samples, including hemes, ISCs, and non-heme iron centers (19). EPR spectroscopy can detect paramagnetic iron centers whereas MB spectroscopy can detect iron in any spin or oxidation state as long as the ^57^Fe isotope is used. MB spectroscopy exclusively detects this isotope since it possesses nuclear spin I = 1/2. The low natural isotopic abundance of ^57^Fe (∼2%) compared to MB-silent isotopes ^56^Fe and ^54^Fe (collectively ∼98% of iron in nature) is typically a disadvantage that lowers spectral intensities. This problem is often minimized by growing cells in media enriched with ^57^Fe. Enrichment is required because the iron concentration in eukaryotic cells is only ca. 200 to 600 μM (20) and MB spectroscopy is inherently insensitive relative to many other methods. With our instruments, obtaining a single interpretable spectrum from samples containing ∼200 μM ^57^Fe required more than a week of continuous data collection.

Despite these difficulties, we report here the successful detection by MB (and EPR) of what is likely to be the endogenous, artifact-free, LFeP in whole intact *Saccharomyces cerevisiae* cells. Such cells contain about 100 iron-containing proteins (21) as well as LFePs in the cytosol (22), mitochondria (7) and vacuoles (9). By harvesting growing cells soon after adding ^57^Fe to the growth media, the LFeP of these cells appears to have been selectively enriched with ^57^Fe while most of the remaining iron in the cell was MB-silent. By harvesting cells at longer times, ^57^Fe moved into vacuoles and mitochondrial/cytosolic/nuclear ISCs. This analysis was reinforced by simulations obtained using a recently published mathematical model (23). Overall, our results suggest that *we have detected the kinetics of iron trafficking, and perhaps labile iron pools, within intact cells for the first time*.

## Results

Our objective was to observe ^57^Fe entering growing WT yeast cells and distributing into vacuoles and mitochondria in a time-dependent manner. Prior to adding ^57^Fe, cells contained a natural isotopic abundance of iron, and thus were effectively invisible by MB (but observable by EPR).

Three iron states of growing yeast cells should be considered. “*Iron-replete*” cells are those grown in media containing iron at a sufficiently high concentration (*e.g.* 40 μM ^57^Fe^III^ citrate) for vacuoles to be fully loaded with iron. Under aerobic conditions, these organelles store iron as high-spin Fe^III^ ions coordinated to polyphosphate chains. Under anaerobic growth conditions, cells appear to store iron in vacuoles as high-spin Fe^II^ (10, 24). “*Iron-sufficient*” cells are those grown in media supplemented with 0 to ∼4 μM Fe^III^ citrate. Minimal medium that is not supplemented with iron contains enough residual iron for cells to grow at normal WT rates. In such cases, vacuoles are partially loaded with iron. “*Iron-deficient*” cells lack sufficient iron to sustain WT growth. However, achieving this state requires adding an iron chelator such as bathophenanthroline sulfonate (BPS) to unsupplemented minimal media. BPS chelates the residual iron in the media that would otherwise allow WT growth. For iron-deficient cells, the severity of growth inhibition depends on the concentration of BPS added. More problematic is that MB spectra of iron-deficient cells cannot be attained because some ^57^Fe must be added to the medium.

### Mössbauer spectrum of respiring cells

**Figure 1:**
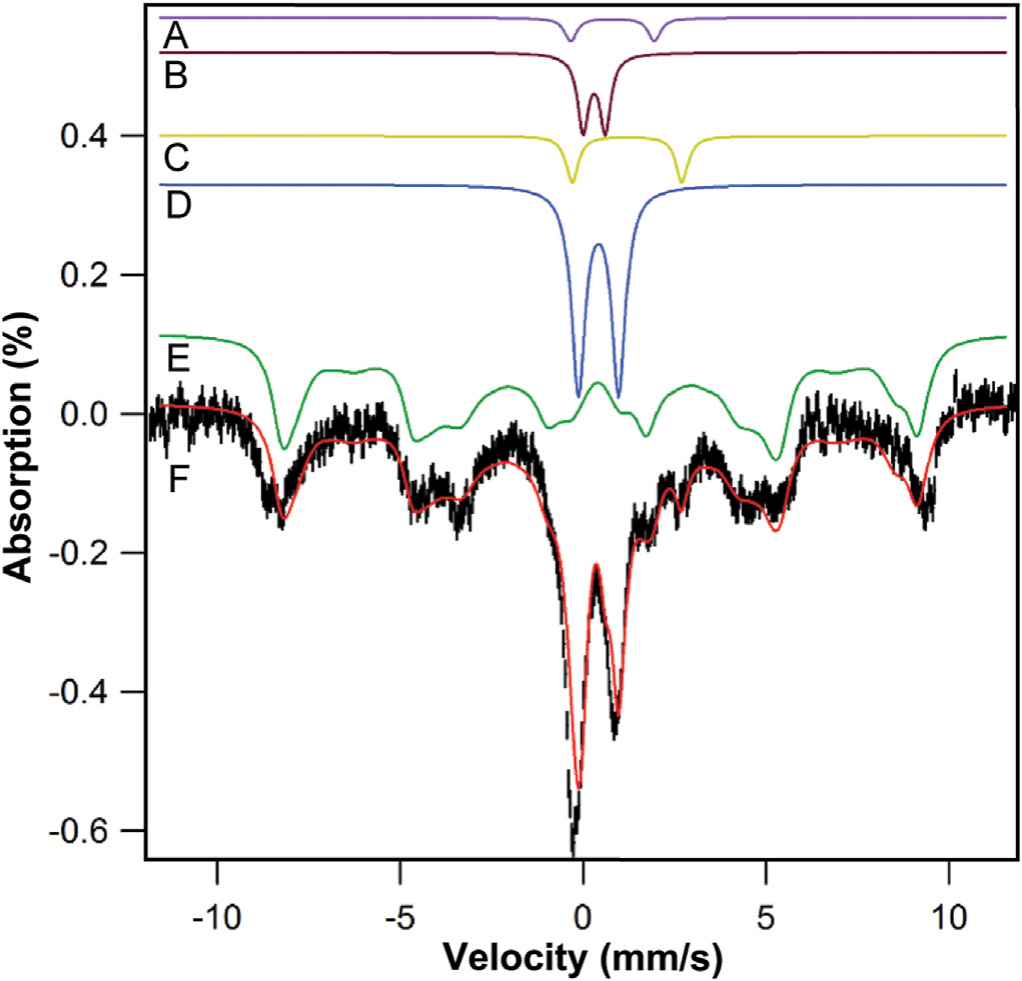
**Mössbauer spectrum of respiring yeast cells grown in minimal medium supplemented with 40 μM**^**57**^**Fe**^**III**^**citrate.***A,purple line*, HS Fe^II^ hemes; *B,maroon line*, S = 0 [Fe_2_S_2_]^2+^ clusters; *C,gold line*, NHHS Fe^II^; *D,blue line*, S = 0 [Fe_4_S_4_]^2+^ clusters and LS Fe^II^ hemes; *E,green line*, NHHS Fe^III^; *F,black hashmarks*, data. The *red line* overlaying the data is the sum of simulated lines A**–**E. In this and all presented MB spectra, a 0.05 T field was applied parallel to the gamma radiation and the sample temperature was 5 to 6 K. Modified from (30).

Iron-replete WT strain W303 respiring yeast cells, grown in media supplemented with 40 μM ^57^Fe citrate and harvested near the end of the exponential growth phase, exhibit MB spectra that can be decomposed into four major features (Fig. 1). Dominating the spectrum is a magnetic feature due to high-spin S = 5/2 Fe^III^. This feature extends from a velocity of −9 mm/s to +10 mm/s and is simulated by the green line in Figure 1*E*, using MB parameters given in Table S1. (See this table for all MB fitting parameters.) Previous studies found that such iron is present in vacuoles (9, 20). This feature represents 65% of overall spectral intensity. Under hypoxic conditions, vacuolar iron becomes reduced to the Fe^II^ state, which causes the intensity of the Fe^III^ features to decline and Fe^II^ features to increase (10).

Iron-replete cells also exhibit a quadrupole doublet with parameters typical of [Fe_4_S_4_]^2+^ clusters and low-spin Fe^II^ hemes (δ = 0.45 mm/s; ΔE_Q_ = 1.15 mm/s) (Fig. 1*D*, blue line); which we call the *central doublet* or CD. Such iron centers are mainly found in mitochondria, cytosol, and nuclei (21).

Iron-replete cells also exhibit a quadrupole doublet with parameters typical of Non-Heme High-Spin (NHHS) Fe^II^ species. This feature, simulated by the gold line in Figure 1*C*, arises from the sum of all LFePs in the cell as well as from NHHS Fe^II^ ions bound to proteins. In this spectrum, it represents only 3 to 5% of overall spectral intensity, but it may represent as much as 30%–50% (10), if cells are grown under hypoxic conditions.

Minor spectral features include a doublet due to low-spin Fe^II^ heme centers (Fig. 1*A*, purple line) and another due to S = 0 [Fe_2_S_2_]^2+^ clusters (Fig. 1*B*, maroon line). Some spectra include additional absorption in that region; it is difficult to assign but often can be simulated by a doublet typical of superparamagnetic Fe^III^ oxyhydroxide nanoparticle aggregates (25).

The following experiments are presented in historical sequence. They have the common objective of detecting the kinetics of iron trafficking in these cells. However, each experiment was designed slightly differently, using the results of earlier experiments to improve later ones.

### Experiment 1

A pre-culture of WT cells was grown in 1 L of respiring media supplemented with 4 μM of natural-isotopic-abundance Fe^III^ citrate (all concentrations final). When OD600 reached ∼1, the culture was used to inoculate a custom-built iron-free glass-and-titanium bioreactor containing 23 L of minimal media. Glycerol/ethanol was used as a nonfermentable carbon source. The exponential growth rate of the cells, called α, was defined as the slope of ln (OD600) *vs*. time. For these cells, α was 0.13 h^−1^, corresponding to ∼65% of the normal WT rate (α = 0.20 h^−1^) (7). This corresponds to a doubling time of ln2/α = 320 min.

When OD600 reached 0.41, a liter of culture was removed from the bioreactor as a control. Immediately thereafter, ^57^Fe^II^ (^57^Fe^III^ citrate reduced with ascorbate) was added to the bioreactor, achieving a final concentration of 40 μM. Another sample was removed about 3 min later (at *t* = 3 min). For this and all other time point samples, including the control (referred to as *t* = 0), a portion was packed by centrifugation into an MB cup and frozen in liquid N_2_. Another portion was similarly packed into an EPR tube and frozen, and a third portion was packed into a plastic screw-top tube for metal analysis. Additional samples were prepared at *t* = 30, 60, 120, 180, and 300 min. The dead time for sample handling was estimated at 5 to 15 min. The large range reflects our inability to establish when metabolic activity in these cells ceased. It certainly declined when cells were separated from growth media, and declined further when centrifuged cells were resuspended in water. Metabolism fully ceased when re-pelleted cells were frozen in liquid N_2_.

We also monitored the growth of the cells during the harvesting period. For *t* = 3, 30, 60, 120, 180, and 300 min samples, OD600 was 0.46, 0.46, 0.46, 0.61, 0.59, and 0.83, respectively. This indicates that the cells initially stopped growing after adding the ^57^Fe, and then started growing again at about the same rate as before the addition (α = 0.13 h^−1^). We cannot explain the hr-long lag period associated with adding iron nor why post-lag iron-replete cells did not grow faster than iron-limited cells. The growth rate throughout the experiment must have been controlled by something other than the iron concentration in the media.

**Figure 2:**
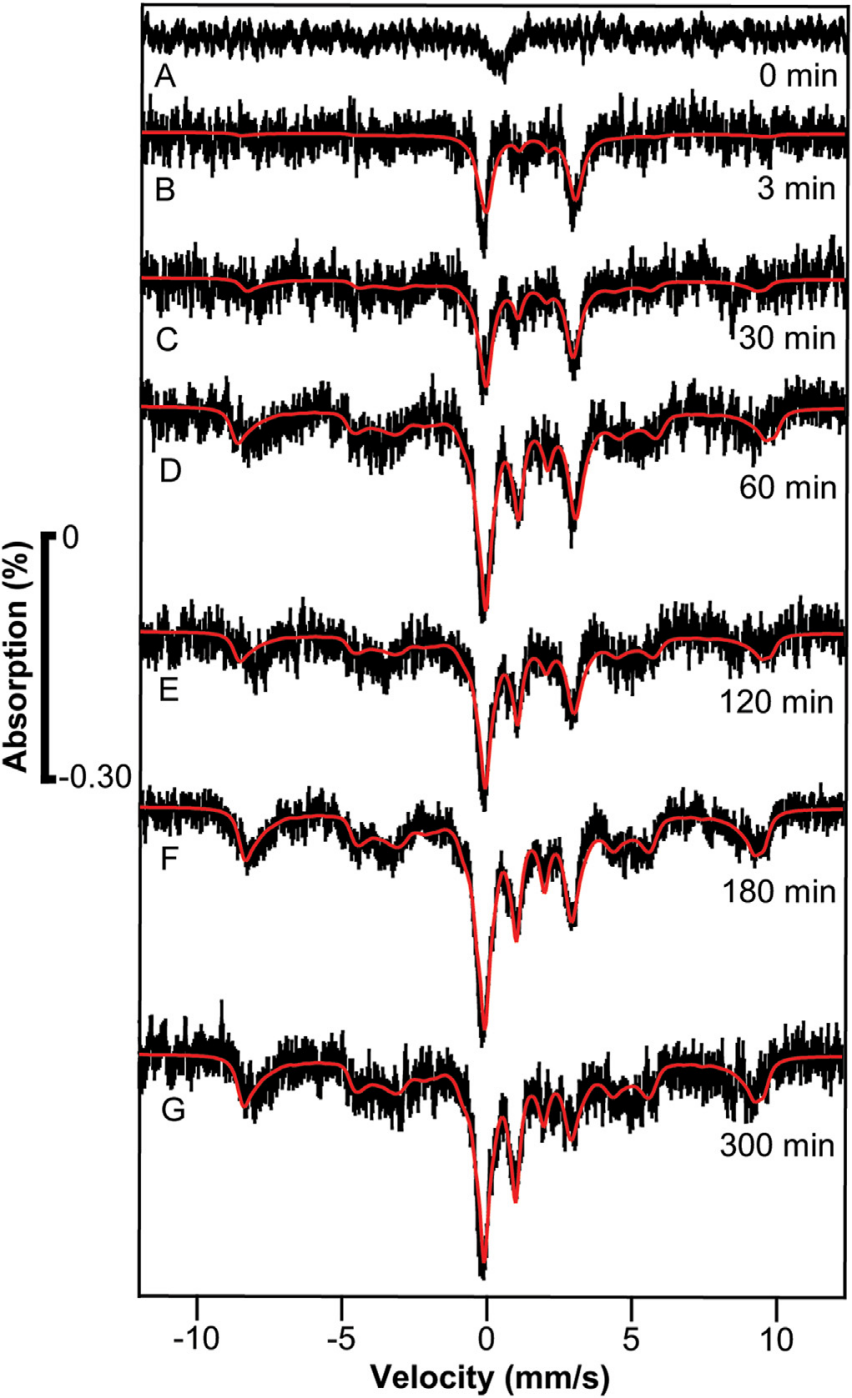
**Mössbauer spectra of**Experiment 1**samples.** Spectra *A****–****G* are of whole cells harvested at the indicated times after ^57^Fe^II^ was added to respiring WT cells, defined as t = 0. Cells were grown with natural-isotopic abundance iron. The control spectrum, which exhibited minor unassigned absorption perhaps from the windows of the instrument, was simulated and subtracted from the raw spectra. Fitting parameters are given in Table S1.

The control sample (Fig. 2*A*, 0 min) exhibited minor absorption near 0 velocity, possibly due to minor contamination on the cryostat window. That background signal was simulated and subtracted from all other spectra in the series. The sample harvested at *t* = 3 min exhibited a single quadrupole doublet (Fig. 2*B*) with parameters (δ = 1.38 mm/s; ΔE_Q_ = 2.87 mm/s; Γ = 0.47 mm/s) typical of NHHS Fe^II^ complexes with 5 to 6 ligands possessing majority O or N donor atoms. The spectral intensity was low because the concentration of ^57^Fe in the cells was low – we estimate 20 to 50 μM. As a result, each spectrum had to be collected for > 250 h to achieve acceptable S/N. Such lengthy collection times were required for virtually all spectra in the study. The lower-than-ideal S/N of the spectra also affected the uncertainties of reported isomer shifts and quadrupole splittings (which we estimate to be ± 0.1 mm/s).

This Fe^II^ doublet did not arise from the ^57^Fe^II^ in the growth medium. ^57^Fe^II^ citrate exhibits a doublet with δ ≈ 1.3 mm/s and ΔE_Q_ ≈ 3 mm/s (14). In Experiment 1, harvested cells were washed with water to remove exogenous ^57^Fe^II^ prior to packing into MB cups such that the concentration of added ^57^Fe^II^ in the interstitial buffer associated with the pelleted whole-cell sample should have been <1 μM. At this concentration, nutrient ^57^Fe^II^ would have been undetectable by MB spectroscopy, by at least an order of magnitude.

The NHHS Fe^II^ doublet was more intense in the MB spectrum of the *t* = 30 min sample (Fig. 2*C*), implying that additional ^57^Fe^II^ had entered the cell. The spectral baseline was not entirely flat, but rather exhibited features suggesting that vacuoles contained NHHS (S = 5/2) ^57^Fe^III^ at a low concentration. A minor peak was present at a velocity of ∼ 1 mm/s (where the high-energy line of the CD would be expected) but the severity of the noise precluded a definitive assignment.

The *t* = 60 min sample exhibited the NHHS Fe^II^ doublet, a more intense NHHS Fe^III^ feature, and a more obvious CD (Fig. 2*D*). Spectra of the *t* = 120, 180 and 300 min samples (Fig. 2, *E*–*G*) were similar, suggesting that the kinetics of iron import and trafficking in respiring yeast cells under the described growth conditions had completed 60 to 180 min after adding ^57^Fe. The 320-min doubling time for these cells suggests that the rate of iron import, trafficking, and metallation were all faster than the rate of cell growth and replication. This implies that the detected changes reflect physiologically relevant trafficking processes occurring within growing cells.

**Figure 3:**
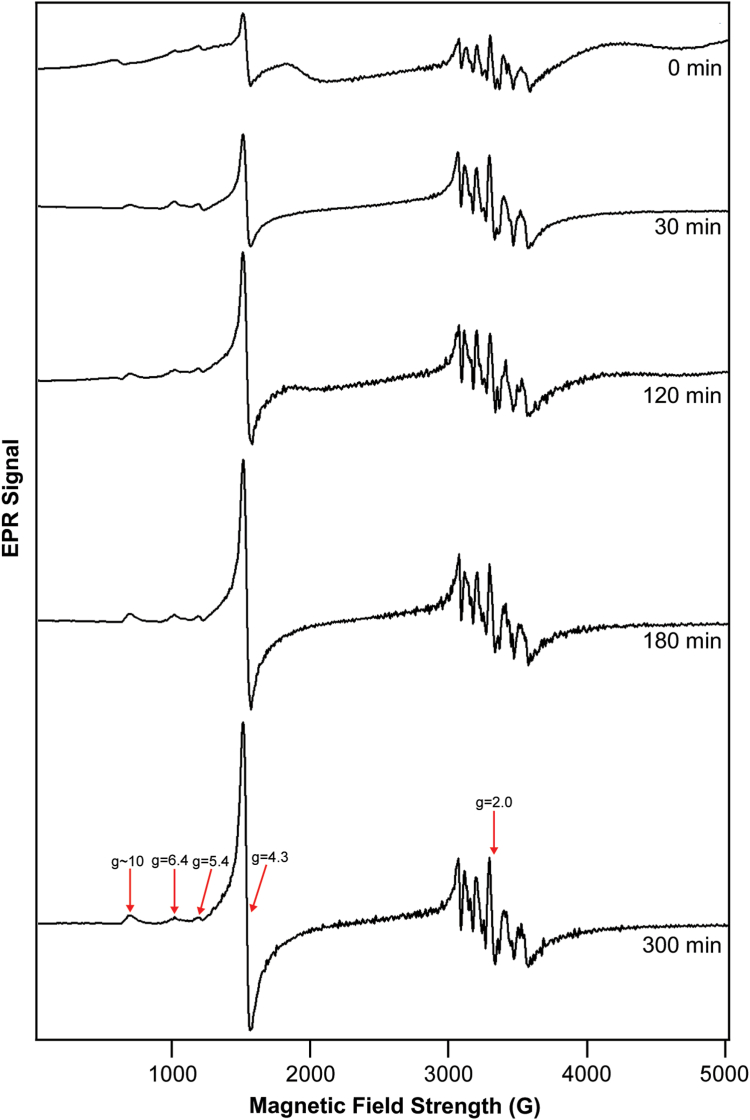
**EPR spectra of**Experiment 1**samples.** Spectra are of matched samples to those of Figure 2 (the t = 60 min spectrum could not be obtained). Spectrometer conditions: microwave frequency, 9.378 GHz; microwave power, 0.2 mW; Modulation amplitude, 5 G. Temperature, 10 K; Sweep time, 335 s.

Corresponding EPR spectra (Fig. 3) were dominated by a hyperfine-split signal in the g = 2 region (at ca. 3350 G) due to cellular Mn^II^ (S = ½; I = 5/2) ions (20). Similar species were detected and characterized by Hoffman and Culotta using ENDOR spectroscopy (18) and by Holmes-Hampton *et al.* using EPR (20). Also evident in the spectra was a g = 4.3 signal (at ∼ 1570 G) previously observed and assigned to the S = 5/2 Fe^III^ ions in vacuoles (20, 24). The nearly invariant intensity of the Mn signal fortuitously served as an internal standard. The absence of a g_ave_ = 1.94 signal indicates that most of the [Fe_4_S_4_] and [Fe_2_S_2_] clusters in the cells are in the diamagnetic 2+ core oxidation state, consistent with the Mössbauer spectra.

The increasing intensity of the Fe^III^ g = 4.3 signal with time after adding ^57^Fe^II^ indicates that the vacuoles were being filled with the added ^57^Fe over the course of 300 min. This result was *qualitatively* consistent with the MB spectra of Figure 2 which suggested that the vacuoles were filled in ≈ 60 min. Both MB and EPR data suggest that the rate of iron trafficking into vacuoles (as Fe^II^, entering *via* the vacuolar importer CCC1) and subsequent oxidation to the Fe^III^ state was either comparable to, or faster than, the growth rate of the cell. We suspect that the EPR results were more accurate, given the poor S/N of the MB spectra and the helpful presence of an internal Mn standard.

Although minor in intensity, the EPR features at g = 6.4 and 5.4 (see arrows in Fig. 3) deserve attention. These features are characteristic of S = 5/2 Fe^III^ hemes with rhombic symmetry. A signal with the same g-values (likely to be the same signal) has been observed in spectra of isolated mitochondria (26). The signal was assigned decades ago to an intermediate oxidation state of cytochrome c oxidase contained in the organelle. In this state, heme a_3_ of the a_3_:Cu_b_ active site is high-spin Fe^III^ whereas Cu_b_ is diamagnetic Cu_b_^I^ (27). In our spectra, the relative intensities of these signals did not increase noticeably with time, implying that the added ^57^Fe was *not* used to assemble this center in this experiment. We suggest that, before adding ^57^Fe, the vacuoles were largely devoid of Fe, but that cytochrome c oxidase in mitochondria was filled with ^56^Fe hemes at normal levels. This is consistent with the cells being Fe-sufficient but not Fe-deficient in this experiment.

We also measured the iron concentration in the harvested cells in Experiment 1 (Table S2). The overall concentration of iron in these cells averaged over the different times collected, was 660 ± 200 μM, similar to previous determinations (20). The cells were clearly not iron-deficient at *t* = 0 but they became enriched in ^57^Fe as a function of time (ca. 2% → 30% → 60%) as ^57^Fe entered the LFeP of the cell and then distributed into vacuoles and mitochondria.

### Experiment 2

In this experiment, we wanted the cells to begin more iron-deficient than they were in the first experiment. This was difficult because MB spectra cannot be obtained unless some ^57^Fe is included in the growth medium, but doing so renders the cells either iron-sufficient or iron-replete. In this experiment, we attempted to do both by adding BPS (25 μM) to the growth medium and then adding ^57^Fe (4 μM) after cells reached a higher OD600 (0.92). Other differences relative to Experiment 1 were that cells were washed more extensively during harvest and that ^57^Fe^III^ citrate was added rather than ascorbate-reduced ^57^Fe^II^. During harvesting, cells were washed in 400 μM BPS + 1 mM ascorbate, and then in water.

A sample was removed just prior to adding ^57^Fe^III^ citrate at *t* = 0. Additional samples were collected at *t* = 3, 23, 43, 63, 93, 123, 183, and 243 min. Inexplicitly, these cells grew more slowly (α = 0.05 h^−1^) than those of Experiment 1. All samples were collected within a single-cell doubling time (∼830 min).

Adding BPS to the growth medium was not a perfect solution to the problem, because the S = 0 ^57^Fe^II^(BPS)_3_ that formed adhered tightly to cells and thus contributed to MB spectra as an unwanted minor-intensity doublet. The doublet was simulated and electronically subtracted from the raw spectra.

**Figure 4:**
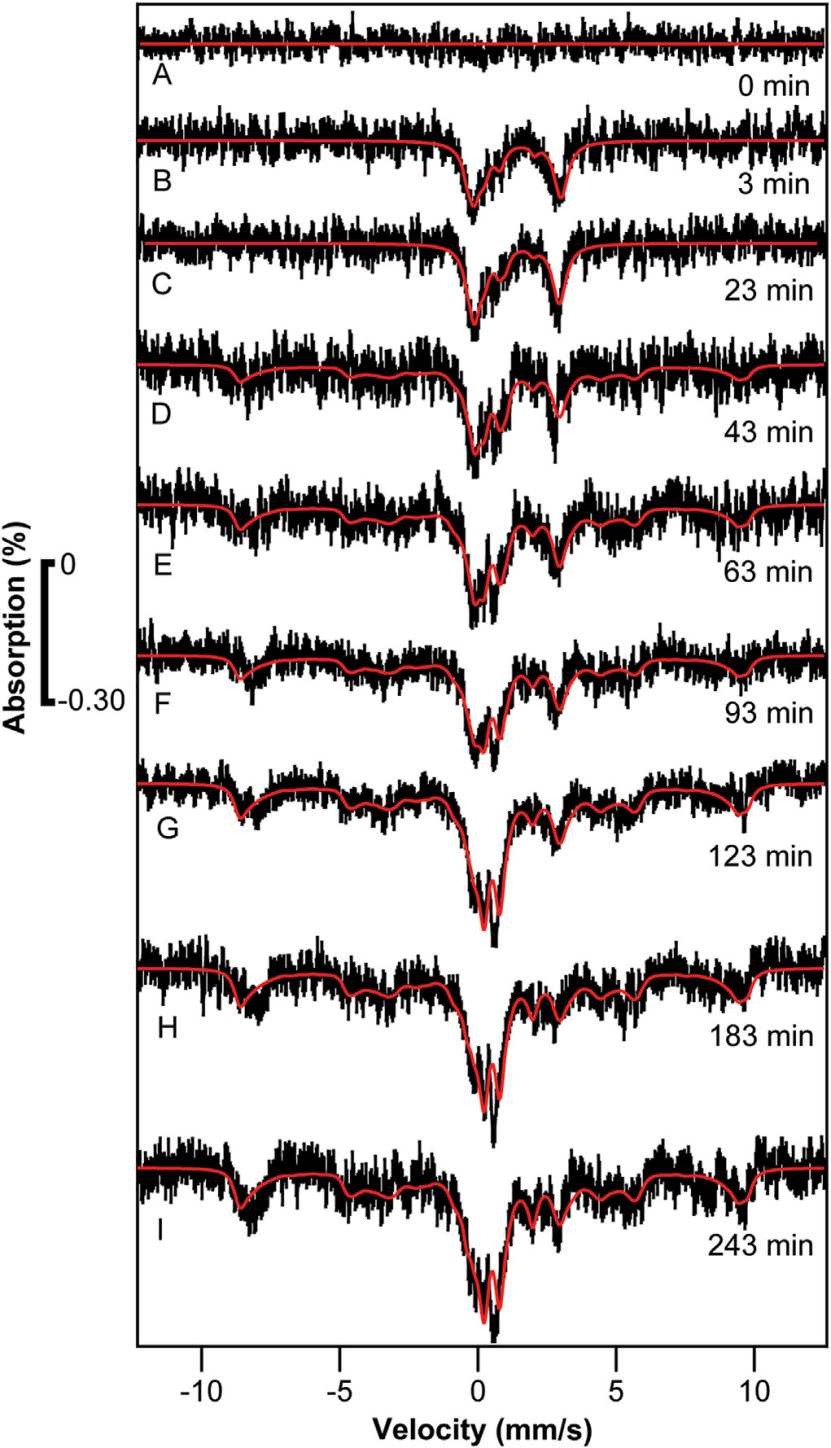
**Mössbauer spectra of**Experiment 2**samples.** Conditions were similar to the spectra of Figure 2. Spectra *A****–****I* were harvested at the indicated times.

The resulting difference spectra (Fig. 4) were remarkably similar to those of Experiment 1. The cells harvested prior to adding ^57^Fe (*t* = 0, Fig. 4*A*) were devoid of signals. The *t* = 3 min sample (Fig. 4*B*) exclusively exhibited a quadrupole doublet due to NHHS Fe^II^. Spectral features due to vacuolar high-spin Fe^III^ and the CD both appeared at *t* = 43 min (Fig. 4*D*), and both were fully developed by 60 to 123 min. Within the uncertainties, the rates of iron import into the cell and the rates of trafficking into vacuoles and mitochondria were similar to those of Experiment 1.

**Figure 5:**
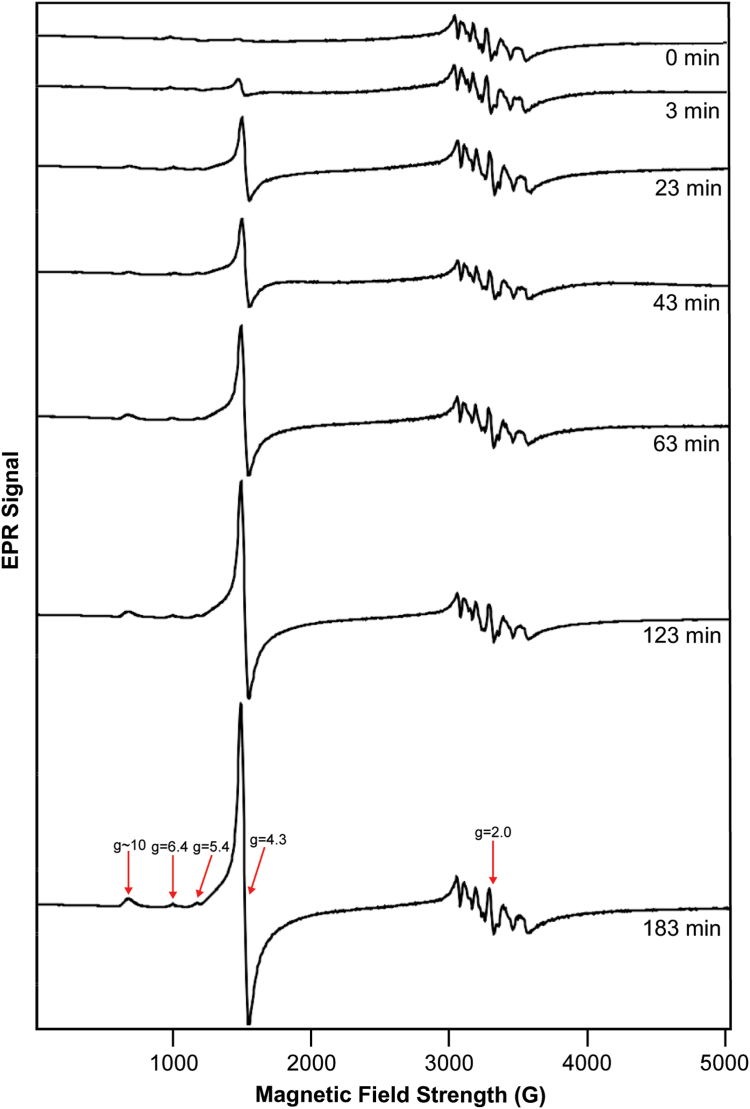
**EPR of**Experiment 2**samples:** Similar to the spectra of Figure 3, but for Experiment 2. Spectra are matched to Figure 4 except for the t = 93 min spectrum which could not be obtained.

The corresponding EPR spectra again revealed an increasing intensity of the g = 4.3 signal (arrow in Fig. 5). The intensity of this signal was greater at *t* = 183 min than at *t* = 123 min, suggesting that iron was being imported into the organelle during that time period. The complete absence of the g = 4.3 signal in the *t* = 0 min sample indicated that the cells were iron-deficient prior to adding ^57^Fe. The rate at which this signal (and the associated feature at g ≈ 10, arrow in Fig. 5) increased was qualitatively consistent with the MB spectra of Figure 4. Like the EPR results of Experiment 1, these spectra also exhibited an Mn-based hyperfine-split signal in the g = 2 region, the intensity of which was largely invariant with time and thus was useful as an internal standard.

The EPR spectra of Experiment 2 also exhibited minor-intensity high-spin Fe^III^ heme signals at g = 5.4 and 6.4 (arrows, Fig. 5) likely arising from an intermediate oxidation state of mitochondrial cytochrome c oxidase. Like the spectra of Experiment 1, the intensities of these signals were approximately invariant with time after adding ^57^Fe. Thus, both experiments suggested that the metallation level of mitochondrial cytochrome c oxidase in these two experiments was unaffected by the added ^57^Fe.

### Experiments 3 and 4

At this point, we focused on establishing the major conclusions of the two previous experiments—namely that the first intracellular ^57^Fe species to be observed was an NHHS Fe^II^ doublet followed at later times by transport into vacuoles and mitochondria. In Experiments 3 and 4, no BPS was added to the media, less media volume was used and fewer time points were collected. Cells were grown in 1.5 L of respiring minimal media (glycerol/ethanol carbon source) prepared with HPW in 3 L nitric-acid-washed plastic baffled flasks. They grew to an OD600 ≈ 1.0 at which time cells from 500 ml of culture were packed into an MB cup (the control). Immediately thereafter, 1 μM of ^57^Fe^III^ citrate (final concentration) was added at *t* = 0. At *t* = 15 min, another 500 ml of culture was spun down into an MB cup. Upon harvesting, cells were poured onto crushed ice to abruptly halt cellular metabolism and shorten the processing dead time. Centrifuged cells were washed with cold EDTA/water and then again with cold water alone. At *t* = 70 min, the remaining 500 ml of cells were similarly spun down into an MB cup. In this experiment, cells grew at α ∼ 0.07 h^−1^, again slower than normal but similar to our other experiments. Experiment 4 was conducted similarly except that the OD600 at which ^57^Fe was added was 1.2 and the final sample was collected at *t* = 75 min.

**Figure 6:**
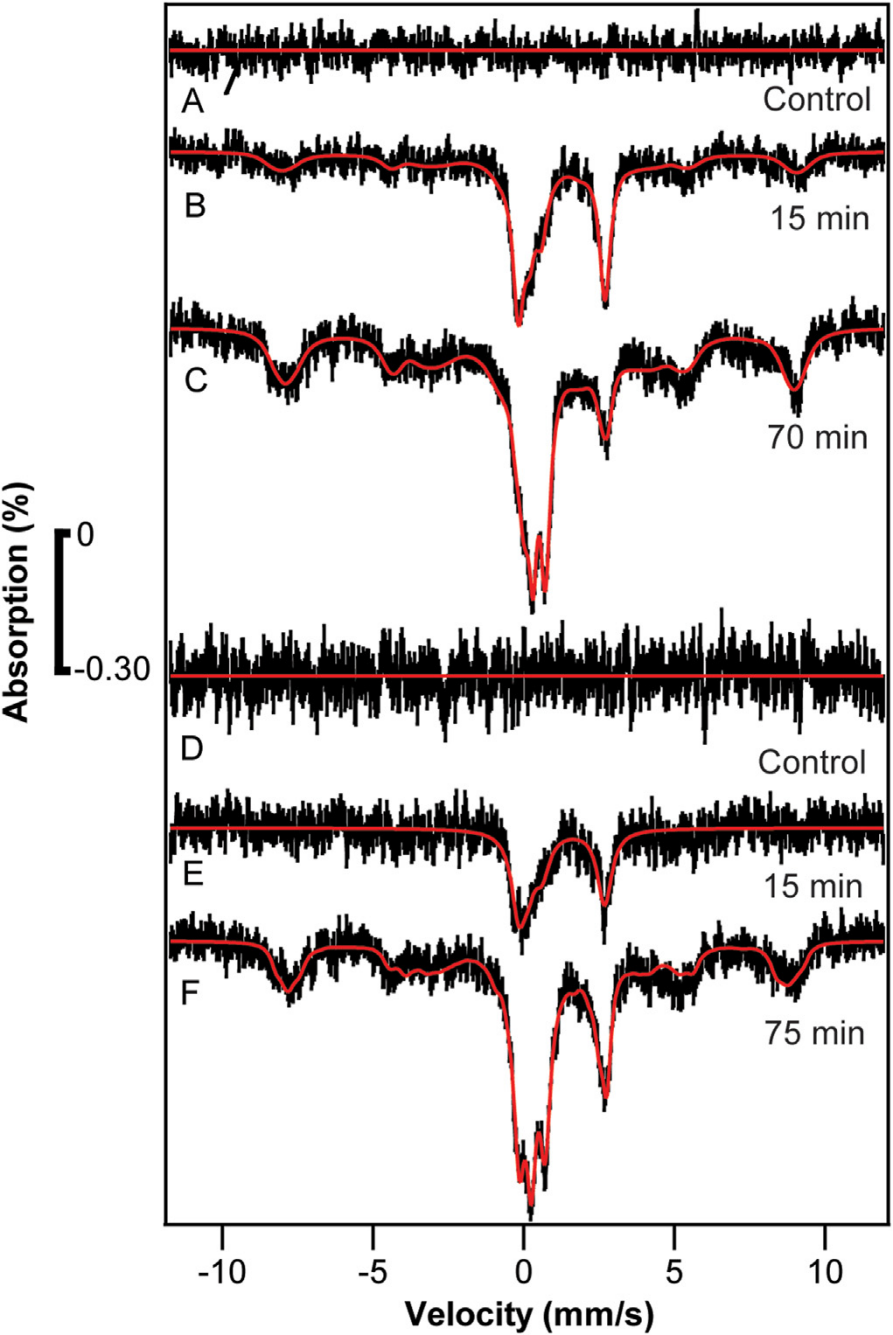
**Mössbauer spectra of**Experiments 3 and 4**samples.** Samples *A−F* were prepared at the indicated times, as described in the text.

In both experiments, the control spectra were devoid of signals (Fig. 6, *A* and *D*) while samples harvested at *t* = 15 min (Fig. 6, *B* and *E*) exhibited an intense NHHS Fe^II^ signal. Minor features in the baseline were likely due to a low concentration of vacuolar Fe^III^.

The *t* = 70 and 75 min samples (Fig. 6, *C* and *F*, respectively) both exhibited a NHHS Fe^II^ doublet, NHHS Fe^III^ magnetic features, and a poorly resolved region in the center of the spectra that fit to the CD and another unassigned doublet. The latter doublet was fitted with parameters of Fe^III^ oxyhydride nanoparticles but other assignments are also possible. The relative intensities of these features were similar between samples but were not identical.

Regardless, we conclude that ^57^Fe^II^ entered the cells in less than 15 min and that some of that iron trafficked into both vacuoles and mitochondria 15 to 75 min after adding ^57^Fe to the media. Interestingly, in these experiments, 1 μM ^57^Fe^III^ citrate in the media proved sufficient for cells to store iron in vacuoles; in previous studies, 10 to 40 μM ^57^Fe were required (20).

### Experiment 5 series

This series of 9 separate experiments was an expansion of the previous two experiments in terms of harvesting times. Cells were grown in either 750 ml (*t* = 5 and 15 min samples) or 1.5 L (all other samples) of media, each in 3 L capacity baffle-flasks. Additional efforts were made to lower the concentration of endogenous iron in the media by using “zero medium” composed of: (a) HPW; (b) iron-deficient yeast-nitrogen-base (YNB) at half-normal concentration; and (c) BPS (10 or 30 μM final concentration), placed in acid-washed flasks. We used half-normal concentrations of YNB after discovering that this was the major source of residual iron in the media (Table S3).

For each experiment of this series, when OD600 of the 100 ml pre-culture was ∼ 1, it was used to inoculate the media in a baffle flask. When the OD600 of the baffle flask equaled 0.30, 11 μM (final concentration) of ^57^Fe^III^ citrate was added (*t* = 0). Aliquots were harvested at *t* = 5 and 15 min.

For the other 7 samples in the series, 4.3 μM ^57^Fe^III^ citrate was added to cultures supplemented with 10 μM BPS at the same OD600. Samples were harvested at *t* = 5, 15, 30, 60, 180, 420, 500, and 600 min (at OD600 = 0.30, 0.31, 0.31, 0.34, 0.45, 0.80, 0.95, and 1.2, respectively). Cells were poured into crushed ice at the indicated times, centrifuged, and resuspended in 50 ml HPW. Samples were washed a second time with HPW, slurries were packed into Mössbauer cups and then frozen in LN_2_ for later analysis.

**Figure 7:**
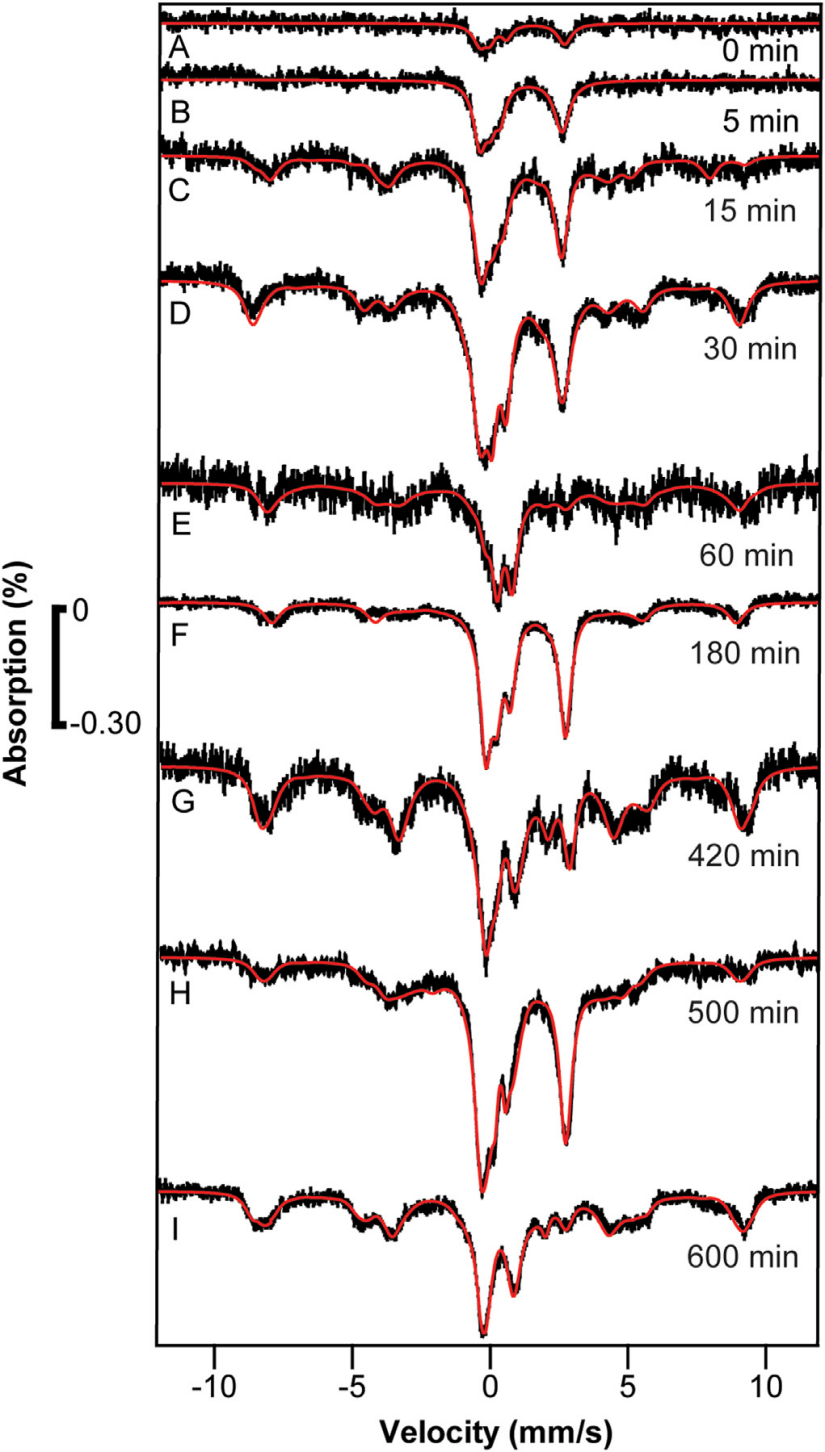
**Mössbauer spectra of**Experiment 5 series**:** Samples *A−I* were prepared at the indicated times, as described in the text.

A control sample was prepared as above at OD600 = 0.30. In this case, cells were harvested prior to adding ^57^Fe^III^ citrate. A slurry of ice-cold cells was incubated with ^57^Fe^III^ for 5 min. Cells were then centrifuged as above, washed, and loaded into a Mössbauer cup and frozen. The spectrum of this control exhibited a minor NHHS Fe^II^ doublet (Fig. 7*A*), implying that the rate of iron import in the cells was rapid (within the dead time of the experiment) even in cold metabolically sluggish cells.

The other samples in this series exhibited MB spectra with greater intensities. The *t* = 5 min sample exclusively exhibited a NHHS Fe^II^ doublet (Fig. 7*B*). The *t* = 15 min sample exhibited a similar doublet but with magnetic features from vacuolar S = 5/2 Fe^III^ evident in the baseline. The spectrum also exhibited unresolved absorption near the low-energy line of the NHHS Fe^II^ doublet (Fig. 7*C*) which probably arose from an emerging CD. The *t* = 30 min sample included three major spectral features, namely the NHHS Fe^II^ doublet, vacuolar S = 5/2 Fe^III^, and again unresolved absorption in the central spectral region (Fig. 7*D*). The *t* = 180 and 500 min samples exhibited similar spectra (Fig. 7, *F* and *H*), with the unresolved absorption simulated with a CD doublet.

The *t* = 60 min sample exhibited similar features except that the intensity of the NHHS Fe^II^ doublet was diminished (Fig. 7*E*). For this sample, packed cells only partially filled the MB cup, explaining the lower spectral S/N. In this spectrum, the S = 5/2 magnetic features were relatively more intense and the unresolved absorption could be simulated with less ambiguity as the CD. The *t* = 420 and 600 min samples exhibited similar spectra (Fig. 7, *G* and *I*).

Spectra from samples *t* > 5 min readily divided into two groups, with *t* = 15, 30, 180, and 500 min composing what we call the *hypoxic* group and the other, with t = 60, 420, and 600 min, composing the *aerobic* group. Previous spectra (10) and mathematical simulations (below) indicate that small differences in the dissolved O_2_ concentration of cell cultures were responsible for these different patterns.

### Mathematical modeling

**Figure 8:**
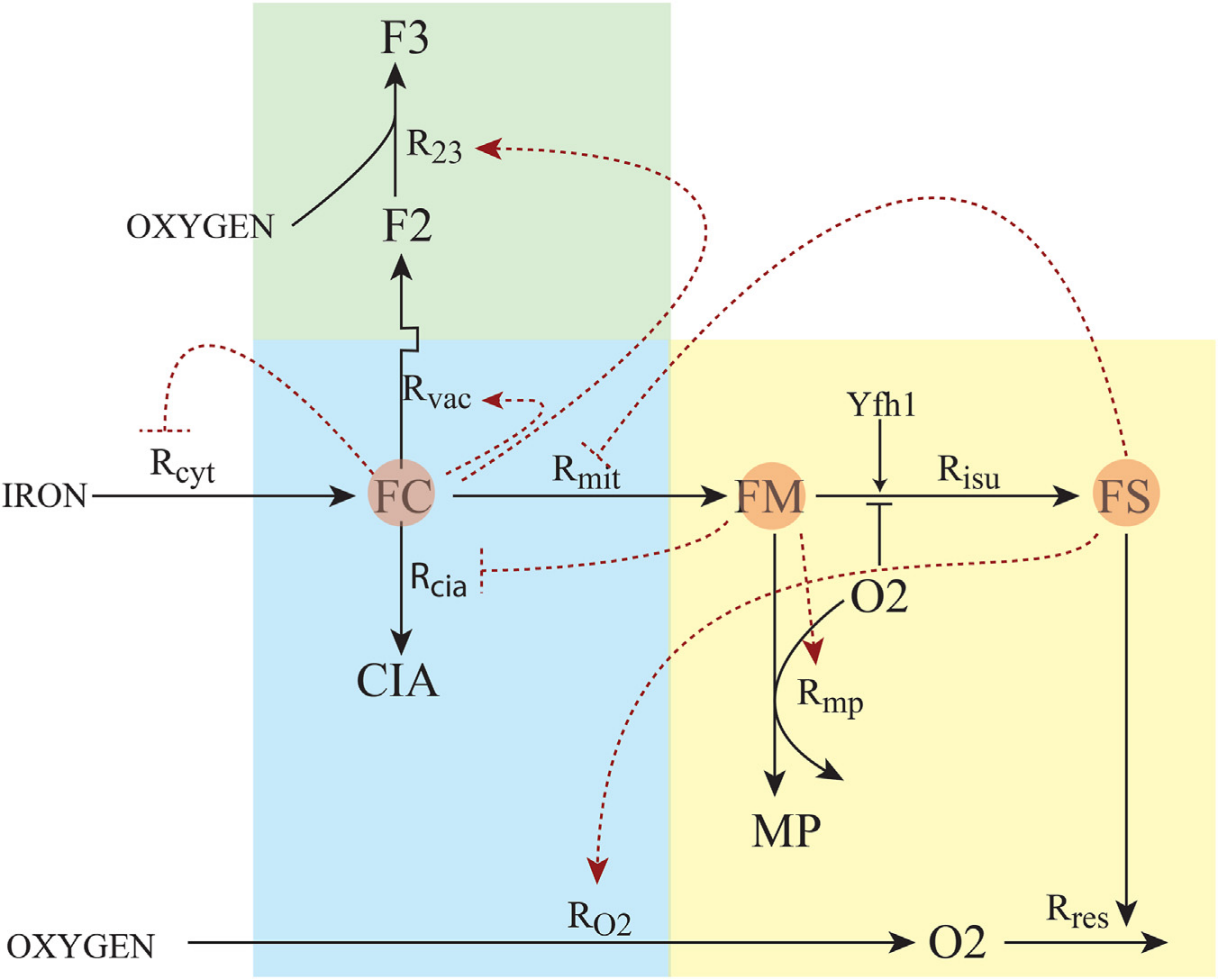
**Biochemical Mechanism used in modeling.***Green, blue*, and *yellow* regions refer to vacuoles, cytosol, and mitochondria, respectively. *Black lines* indicate trafficking reactions, with rates *R*_*xxx*_. *Red shaded circles* indicate sensed species. *Dashed red lines* indicate regulatory processes. Such lines terminating in *short perpendicular lines* indicate inhibition by the sensed species; those terminating in *arrows* indicate stimulation. See the text and (23) for details.

Based on earlier models (10) we recently developed an ordinary-differential-equations (ODE)-based mathematical model describing the kinetics of iron trafficking and regulation in a respiring yeast cell (23). As the *in silico* cell grows, it can also transition from an iron-deficient to an iron-replete state. Thus, the program could be used to simulate the kinetic experiments described above. This provided an opportunity to test the model against experimentally obtained data. To ensure a valid test, no aspect of the model, including the mechanism of Figure 8 and the fitting parameters, was altered relative to those published (23), including the growth rate of the cells.

The nine reactions and 10 components that compose the model are illustrated in Figure 8. Nutrient **IRON** enters the cell and becomes the cytosolic labile Fe^II^ pool, component **FC** (components are introduced in bold). Iron from this pool can enter mitochondria, forming the labile Fe^II^ pool in that organelle, called **FM**. FC can also enter vacuoles, forming the vacuolar labile Fe^II^ pool, component **F2**. Upon oxidation, this becomes the Fe^III^ pool, **F3** (9). Alternatively, FC can react to form **CIA** iron which represents all iron in the cell apart from those species just mentioned. Informally, this component is considered to arise from S = 0 [Fe_4_S_4_]^2+^ clusters in the cytosol and nucleus (21). In mitochondria, FM can be used to form iron-sulfur-clusters and heme groups, collectively represented by component **FS**. Nutrient **OXYGEN** enters the cell and becomes **O2**. Under healthy conditions in which the rate of respiration (catalyzed by FS) is fast, most O2 is reduced to water, leaving the matrix region hypoxic. Under a diseased state in which the rate of respiration is slowed, more O2 enters the matrix where it reacts with FM to generate nanoparticles **MP**. This aspect was not a focus of the current study which exclusively involves healthy WT cells transitioning from the iron-sufficient to iron-replete state. Neither was the regulatory network (red dashed lines) that controls iron trafficking a current focus. Interested readers are referred to (23) for details.

The modest S/N ratios and limited resolution of species obtained experimentally allowed only three spectral features to be modeled for this study including: (a) a NHHS Fe^II^ doublet; (b) S = 5/2 Fe^III^ magnetic features; and (c) the CD. Relating these spectral features to the components of the model required grouping model components. The NHHS Fe^II^ doublet was presumed to include contributions of FC, FM, and F2. The S = 5/2 Fe^III^ features were assumed to arise exclusively from F3, and the CD was assumed to arise from both FS and CIA.

Another complication was that simulations provided *local* concentrations of each component, as would be present in isolated cytosol, mitochondria, or vacuoles. In contrast, MB intensities reflect percentage contributions of overall cellular concentrations. Thus, simulations were converted to cellular concentrations by multiplying local concentrations by fractional volumes, assumed to be 0.8, 0.1 and 0.1, respectively for the three cellular compartments listed above.

Another complication was that simulations included *all* iron in the cell (isotopes not distinguished) whereas MB spectra are sensitive to only ^57^Fe. This difference was dealt-with as follows. Simulations began in the iron-deficient state (called the *D* state in (23)) by using the associated steady-state concentrations as obtained when the nutrient iron concentration [IRON] is fixed at 1 μM. However, in the current simulations, starting at *t* = 0, [IRON] was set to 40 μM, as is used for the iron-replete *W* state. This simulates the injection of ^57^Fe into growing unenriched cultures. With time, component concentrations evolved to the steady-state concentrations of the *W* state. However, only the *difference* in simulated concentrations ([W]_steady-state_ – [D]_t_) would reflect the contribution of the added ^57^Fe and only that difference would have been MB detectable. Thus, only those differences are plotted in Fig. S1.

A final complication arose from our inability to achieve the iron-deficient *D* state in these experiments (23). To account for this, initial concentrations were assumed to be half of the steady-state concentrations previously associated with the *W* state.

As mentioned above, differences observed for some longer-time samples of the Experiment 5 series were most likely due to minor differences in the dissolved [O2] in the growing cultures. To simulate fully aerobic conditions, [O2] was set to 100 μM whereas for hypoxic simulations, it was set to 50 μM.

**Figure 9:**
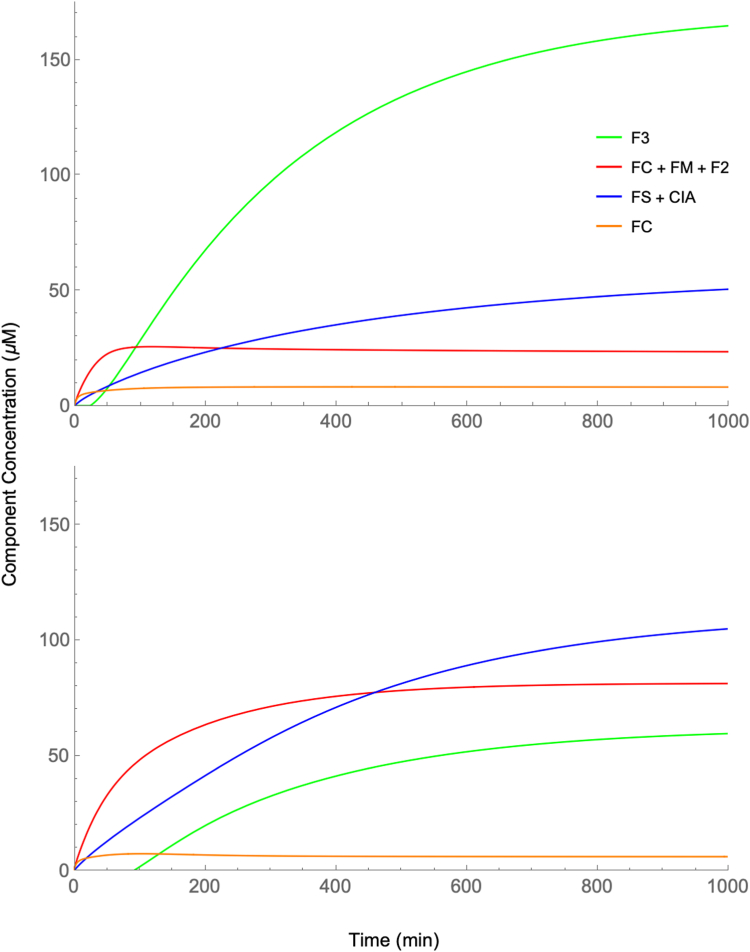
**Simulated time-dependent changes in the concentrations of major iron-containing species in whole healthy WT cells as they transition from an iron-sufficient to an iron-replete state:** Simulations show concentrations of ^57^Fe for the three major MB spectral features including nonheme high-spin Fe^III^ in vacuoles (*green*), labile NHHS Fe^II^ pools combined (*red*), and central doublet reflecting primarily [Fe_4_S_4_]^2+^ clusters and low-spin Fe^II^ hemes (*blue*). *Top panel*, aerobic conditions (100 μM dissolved O2); *bottom panel*, hypoxic conditions (50 μM O2). Although not specifically detectable by MB, the simulated concentration of the cytosolic labile Fe^II^ pool (*orange*) is predicted to be the first species to develop. The kinetic parameters of the W state were presumed throughout. At the start of the simulation (t = 0), component concentrations mid-way between an iron-deficient state and the iron-replete state were assumed, and nutrient [IRON] was set to 40 μM. The cell transitions to the iron-replete state in ca. 1000 min. The major difference between aerobic and hypoxic simulations was the dominance of F3 in the former and {FC + FM + F2} in the latter. For both plots, the initial concentrations for each component groups have been excluded to simulate results obtained by MB spectroscopy. [F3] decreased slightly during the first 10 to 90 min after IRON addition, possibly due to the auto-regulatory mechanism employed (23). Since difference-concentrations are plotted, this led to the appearance of a negative concentration during that period. From the MB perspective, any initial decline of vacuolar iron would have been undetectable and so during that region, minor negative concentration differences (representing < 4% of [F3] at t = 500 min) were redefined as being 0.

The resulting simulations are plotted in Figure 9. The upper panel refers to fully aerobic simulations while the lower panel refers to hypoxic conditions. Both predict that the first MB feature to develop should be NHHS Fe^II^ (solid red lines), as observed experimentally. This feature in the model was obtained by summing all labile iron pools in the cell, namely [FC] + [FM] + [F2]. Although MB spectra cannot distinguish each pool, simulations predict that the cytosolic labile Fe^II^ pool (orange line) should dominate at early times (*t* = 0 → 10 min) followed at *t* ≈ 20 → 1000 min by the growth of vacuolar S = 5/2 Fe^III^ ([F3]) and the CD ([FS] + [CIA]). A similar pattern was observed experimentally but at a faster rate. We could not quantify changes more precisely due to limited spectral S/N ratios and the uncertain dead time required for sample harvesting.

Simulations that assumed a lower [O2] (lower panel) exhibited more intense high-spin Fe^II^ relative to S = 5/2 Fe^III^ and to CD features (lower plot), similar to the pattern observed in the hypoxic group spectra and to previous spectra of the hypoxic state (10). Simulations that assumed higher [O2] (upper panel) were similar to the aerobic group spectra *e.g.* the spectrum in Figure 1.

In summary, the simulations reproduced our experimental results with approximate fidelity. They predicted, as observed, that the labile Fe^II^ pools develop faster than mitochondrial and/or vacuolar iron, and that mitochondrial and vacuolar iron develop approximately simultaneously rather than sequentially. However, the changes predicted by simulations were slower than observed experimentally.

## Discussion

**Figure 10:**
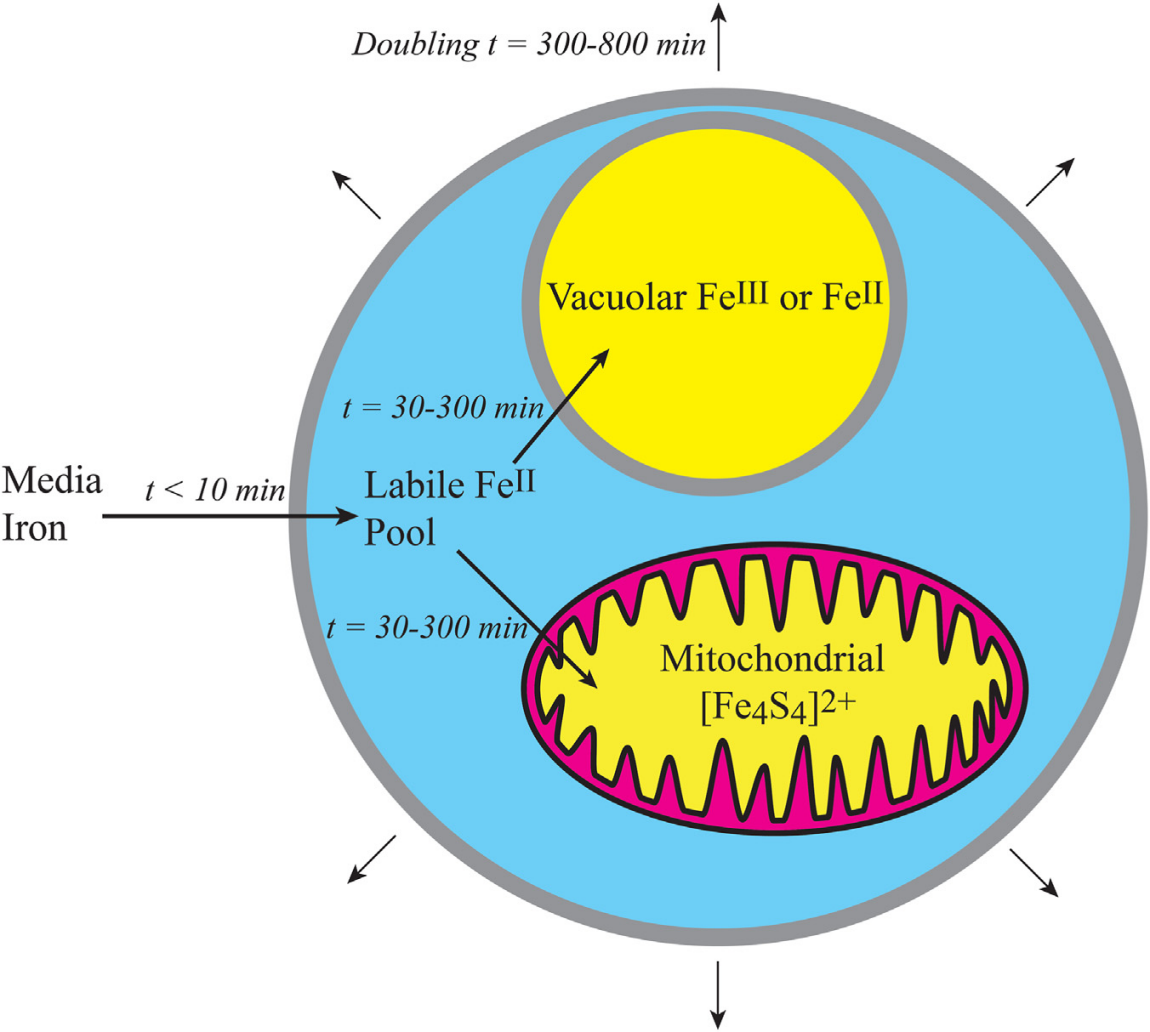
**Summary model.** This study revealed time-dependent changes in MB and EPR spectra of growing yeast cells in a culture to which ^57^Fe was added (at t = 0) and cells were harvested at different times after addition. Our results suggest that the nutrient iron enters the cell quickly (in less than 10 min) and becomes incorporated into the labile Fe^II^ pools. At a slower rate (30–300 min), a portion of that iron is trafficked into vacuoles (and stored as Fe^III^ or Fe^II^) and into mitochondria for use in building iron-sulfur clusters and hemes. These rate estimates are for cells growing with a doubling time of 300 to 800 min.

Our results provide evidence for the basic trafficking model that we and many others (28, 29) have assumed for years (Fig. 10). Accordingly, iron from the media initially enters the cell and is incorporated in the LFeP. Then iron from this pool is distributed into other cellular compartments, mainly mitochondria and vacuoles. Previous evidence indicates that the LFeP consists of a group of NHHS (S = 2) Fe^II^ complexes (see (14) and references therein). Both our experimental results and simulations suggest that the NHHS Fe^II^ quadrupole doublet observed by MB at early times after adding ^57^Fe to growing cultures arose from the *endogenous* LFePs in yeast cells.

In all previous related experiments, endogenous labile pools were either destroyed upon detection (by chelator probes), or they could have been contaminated by artifacts generated during cell lysis (by LC-ICP-MS chromatography). In this study, cells remained intact, and so their labile iron pools should not have been destroyed or contaminated.

This first glimpse of these intact labile pools, as characterized by the MB parameters associated with the early-developing NHHS Fe^II^ doublet, indicates that the Fe^II^ complexes which compose these pools have 5 or 6 O/N donor ligands. The parameters associated with this doublet were within the error of what we observed for Fe^II^ bound to ATP and/or to citrate (δ ≈ 1.3 mm/s; ΔE_Q_ ≈ 3.0 mm/s) (14). We caution that there are many Fe^II^ coordination complexes with 5 or 6 O/N ligands that exhibit similar Mössbauer parameters, and none can be excluded or included based on our data. However, they *can* exclude NHHS Fe^II^ coordination complexes dominated by four sulfur donors in a rubredoxin-like site.

Our results also show that nutrient iron in the growth medium becomes imported into cells *quickly* (<ca. 10 min); this iron likely contributes to the cytosolic LFeP. The concentration of the LFeP in the cytosol of yeast is estimated at 10 to 50 μM (21), and this is consistent with the spectral intensities observed here for the NHHS Fe^II^ doublet in aerobic cells. At an exponential growth rate of α = 0.1 h^−1^, a cell importing 1 μM/min of iron would contain 10 μM of imported iron in 10 min (the same ballpark as we observed for the LFePs) and ca. 400 μM iron at the time of doubling. The calculated concentration of cellular iron measured here and elsewhere (20) is in this range.

In a second slower step, some LFeP moves to the vacuoles for storage, and to the mitochondria where they become assembled in to iron-sulfur-clusters and heme centers. Both processes required 30 to 300 min under the conditions examined. Our data cannot distinguish the rate of import into vacuoles vs. mitochondria; both processes occur at approximately similar rates. The corresponding rates generated by our simulations were slower than observed. Our objective in comparing model simulations with our experimental results was to critically test the model; overall the model did well qualitatively, but reaction rates will need to be adjusted.

We were initially surprised that the vacuoles loaded with Fe^III^ when only 4.3 μM ^57^Fe was added to the growth medium. In previous studies, ∼40 μM Fe added to the growth medium was needed to fill vacuoles with iron (20), and adding 10 μM Fe filled vacuoles partially. However, in those previous studies in which vacuoles were largely empty, cells were grown in the presence of a chelator, a more severe Fe-limited condition than used here. Also unexpected was the large variability in the relative intensity of the NHHS Fe^II^ doublet at long times. Such differences in the redox state of vacuolar iron depend on the oxygenation rate of the cells during growth.

The spectroscopy-based approach introduced here to detect and characterize the LFeP in cells is an alternative to chelator-based and chromatography-based methods. Each approach offers different advantages and disadvantages, such that a combination of them is probably the best current strategy for characterizing LFePs. One advantage of the spectroscopy-based approach is the possibility of selectively enriching the LFeP with ^57^Fe (by harvesting cells at short times after adding the isotope). In principle, it may be possible to demonstrate by LC-ICP-MS that the doublet observed by Mössbauer originates from these pools. We are currently attempting to show this.

## Experimental procedures

The media of Experiments 1 and 2 consisted (per L) of 3% glycerol (Fisher Bioreagents), 1% ethanol (KOPTEC), 1.7 g YNB without ammonium sulfate, copper sulfate, or ferric chloride (MP Biomedicals), 5 g ammonium sulfate (Fisher Chemical), 50 mg adenine hemisulfate dihydrate (MP Biomedicals), 20 mg L-histidine (MP Biomedicals), 20 mg uracil (MP Biomedicals), 100 mg L-leucine (MP Biomedicals), 6 ml of 8 g/L tryptophan, and 1 μM CuSO_4_. The growing culture was sparged with air and stirred as rapidly as possible without vortexing. Ascorbic acid (1 mM final) was added to a stock solution of ^57^Fe^III^ citrate; previous MB experiments showed that this reduced the iron to Fe^II^.

Cells were pelleted by centrifugation at 5000*g* for 5 min (Sorvall Lynx 6000; Thermo Scientific). Suspensions were packed into Mössbauer cups by centrifugation at 10,000 RPM for 5 min using a Beckman Coulter Optima L-90K ultracentrifuge (SW32Ti rotor) and stored in liquid N_2_. EPR samples were packed into quartz tubes (Wilmad) by slow-speed centrifugation (Lynx) using a modified rotor that could accommodate 185 mm long, 4 mm diameter EPR tubes.

For metal analysis, cells were pelleted in EPR tubes and a line was drawn at the pellet:liquid interface. Cells were quantitatively transferred into a 15 ml Falcon tube, and the now empty EPR tube was filled with water to the line and the mass of the water was determined. The volume of the pellet was determined assuming 0.997 g/ml for the density of water. The pellet transferred to the Falcon tube was digested with two parts TMG nitric acid to one part sample at 90 °C for 12 to 16 h. Three samples were prepared from each digest, and samples were diluted to match the concentration of nitric acid to the standards.

The same media was used for Experiments 3 and 4. Flasks were acid-washed before inoculation and shaken in an orbital incubator shaker (Amerex Instruments Inc.) at 30 °C. Baffle-flasks (TriForest) were acid-washed by filling with 3 L of 5% TMG nitric acid (Fisher Chemical), also prepared using HPW. After sitting a day, flasks were rinsed repeatedly with HPW and allowed to dry with caps loosely attached. Glycerol, ethanol, tryptophan, CuSO_4_, and BPS were added after autoclaving and cooling to RT.

Zero medium was used in Experiment 5. This consisted of HPW from a Savillex DST4000 sub-boiling still, starting with house-distilled and deionized water. Other differences relative to the previous media were 0.85 g/L of YNB w/o ammonium sulfate, copper sulfate, or ferric chloride, 2.5 g/L ammonium sulfate, and either 10 or 30 μM BPS (Acros Organics). YNB and ammonium sulfate contained most of the contaminating iron (90 and 30 nM Fe, respectively) so half-normal concentrations were used. Doing so had no effect on the growth rate (Fig. S2) using minimal media.

MB spectra were collected on an MS4 WRC spectrometer (SEE Co) at 5 to 6 K and 0.05 T, with the magnetic field applied parallel to the radiation. An α-iron foil was used for RT calibration. MB simulations were obtained using WMOSS software. The mathematical model was simulated using Wolfram Mathematica. X-band EPR spectra were collected on a Bruker Elexsys E500 spectrometer.

## Data availability

All data are contained within the manuscript and SI.

## Supporting information

This article contains supporting information.

## Conflict of interest

The authors declare that they have no conflicts of interest with the contents of this article.

## References

[1] Outten C.E., Albetel A.N. (2013). Iron sensing and regulation in *Saccharomyces cerevisiae*: ironing out the mechanistic details. Curr. Opin. Microbiol..

[2] Lill R., Dutkiewicz R., Freibert S.A., Heidenreich T., Mascarenhas J., Netz D.J. (2015). The role of mitochondria and the CIA machinery in the maturation of cytosolic and nuclear iron-sulfur proteins. Eur. J. Cell Biol..

[3] Muckenthaler M.U., Rivella S., Hentze M.W., Galy B. (2017). A red carpet for iron metabolism. Cell.

[4] Ganz T., Nemeth E. (2015). Iron Homeostasis in host defense and inflammation. Nat. Rev. Immunol..

[5] Tang Z.M., Zhao P., Wang H., Liu Y.Y., Bu W.B. (2021). Biomedicine meet Fenton chemistry. Chem. Rev..

[6] Breuer W., Epsztejn S., Cabantchik Z.I. (1996). “Dynamics of the cytosolic chelatable iron pool of K562 cells. FEBS Lett..

[7] Moore M.J., Wofford J.D., Dancis A., Lindahl P.A. (2018). (2018) Recovery of Mrs3ΔMrs4Δ *Saccharomyces cerevisiae* cells under iron-sufficient conditions and the role of Fe_580_. Biochemistry.

[8] Holmes-Hampton G.P., Miao R., Garber-Morales J., Guo Y.S., Münck E., Lindahl P.A. (2010). A nonheme high-spin ferrous pool in mitochondria isolated from fermenting *Saccharomyces cerevisiae*. Biochemistry.

[9] Nguyen T.Q., Dziuba N., Lindahl P.A. (2019). Isolated *Saccharomyces cerevisiae* vacuoles contain low-molecular-mass transition-metal polyphosphate complexes. Metallomics.

[10] Fernandez S., Wofford J.D., Shepherd R.E., Vali S.W., Dancis A., Lindahl P.A. (2022). Yeast cells depleted of the frataxin homolog Yfh1 redistribute cellular iron: studies using Mössbauer spectroscopy and mathematical modeling. J. Biol. Chem..

[11] Klausner R.D., Rouault T.A., Harford J.B. (1993). Regulating the fate of mRNA: the control of cellular iron metabolism. Cell.

[12] Carter K.P., Young A.M., Palmer A.E. (2014). Fluorescent sensors for measuring metal ions in living systems. Chem. Rev..

[13] Lindahl P.A., Moore M.J. (2016). Labile Low-Molecular-Mass metal complexes in mitochondria: trials and tribulations of a burgeoning field. Biochemistry.

[14] Brawley H.N., Kreinbrink A.C., Hierholzer J.D., Vali S.W., Lindahl P.A. (2023). The labile iron pool of isolated *Escherichia coli* cytosol likely includes Fe-ATP and Fe-citrate but not Fe-glutathione or aqueous Fe. J. Am. Chem. Soc..

[15] Hider R.C., Kong X. (2011). Glutathione: a key component of the cytoplasmic labile iron pool. Biometals.

[16] Hider R.C., Kong X. (2013). Iron speciation in the cytosol: an overview. Dalton Trans..

[17] Hider R.C., Aviles M.V., Chen Y.-L., Latunde-Dada G.O. (2021). The role of GSH in intracellular iron trafficking. Int. J. Mol. Sci..

[18] McNaughton R.L., Reddi A.R., Clement M.H., Sharma A., Barnese K., Rosenfeld L. (2010). Probing *in vivo* Mn^2+^ speciation and oxidative stress resistance in yeast cells with electron-nuclear double resonance spectroscopy. Proc. Natl. Acad. Sci. U. S. A..

[19] Münck E., Surerus K.K., Henrich M.P. (1993). Combining Mössbauer spectroscopy with integer spin electron paramagnetic resonance. Methods Enzymol..

[20] Holmes-Hampton G.P., Jhurry N.D., McCormick S.P., Lindahl P.A. (2013). Iron content of *Saccharomyces cerevisiae* cells grown under iron-deficient and iron-overload conditions. Biochemistry.

[21] Lindahl P.A., Vali S.W. (2022). Mössbauer-based molecular-level decomposition of the *Saccharomyces cerevisiae* ironome, and preliminary characterization of isolated nuclei. Metallomics.

[22] Nguyen T.Q., Kim J.E., Brawley H.N., Lindahl P.A. (2020). Chromatographic detection of low-molecular-mass metal complexes in the cytosol of *Saccharomyces cerevisiae*. Metallomics.

[23] Thorat S., Walton J.R., Lindahl P.A. (2023). A kinetic model of iron trafficking in growing Saccharomyces cerevisiae cells; applying mathematical methods to minimize the problem of sparse data and generate viable autoregulatory mechanisms. PLoS Comput. Biol..

[24] Cockrell A.L., Holmes-Hampton G.P., McCormick S.P., Chakrabarti M., Lindahl P.A. (2011). Mössbauer and EPR study of iron in vacuoles from fermenting *Saccharomyces cerevisiae*. Biochemistry.

[25] Miao R., Kim H., Koppolu U.M.K., Ellis E.A., Scott R.A., Lindahl P.A. (2009). Biophysical characterization of the iron in mitochondria from Atm1p-depleted *Saccharomyces cerevisiae*. Biochemistry.

[26] Hudder B.N., Morales J.G., Stubna A.A., Münck E., Hendrich M.P., Lindahl P.A. (2007). Electron paramagnetic resonance and Mössbauer spectroscopy of intact mitochondria from respiring Saccharomyces cerevisiae. J. Biol. Inorg. Chem..

[27] Beinert H., Shaw R.W. (1977). On the identity of high-spin heme components of cytochrome c oxidase. Biochim. Biophys. Acta.

[28] Lin H.L., Li L.T., Jia X., Ward D.M., Kaplan J. (2011). Genetic and biochemical analysis of high iron toxicity in yeast. J. Biol. Chem..

[29] Hassett R.F., Romeo A.M., Kosman D.J. (1998). Regulation of high-affinity iron uptake in the yeast *Saccharomyces cerevisiae*. J. Biol. Chem..

[30] Lindahl P.A., Khalimonchuk O. (2024). Iron Metabolism.

