# Supplementary material for: Mössbauer and EPR detection of iron trafficking kinetics and possibly labile iron pools in whole Saccharomyces cerevisiae cells
Source: J Biol Chem. 2024 Aug 22;300(9):107711. doi: 10.1016/j.jbc.2024.107711 (PMC11422575; doi:10.1016/j.jbc.2024.107711)

**Supporting Information for Delanoy et al.** Mössbauer and EPR detection of iron trafficking kinetics and possibly labile iron pools in whole *Saccharomyces cerevisiae* cells

Table of Contents:

Table S1: Mössbauer parameters using in spectral fitting.

Table S2: Iron concentrations in cells from Experiment 1

Table S3: Iron concentration of media components.

Figure S1: Simulation plots of changes in component concentrations as an iron-deficient yeast cell converts into an iron-replete cell after adding 40 µM [IRON] to the growth media.

Figure S2: Absence of an effect of using half-normal concentrations of YNB and ammonium sulfate on growth rate.

**Table S1: Mössbauer parameters used in simulations:**

| Figure # | Component Parameters (Ave) and Fractional Area | | | | |
| --- | --- | --- | --- | --- | --- |
|  | NHHS Fe^III^ Sextet  S = 5/2  δ = 0.55 mm/s  ΔE_Q_ = 0.34±0.08 mm/s  D = -0.08±0.02 cm^-1^  E/D = 0.33  η = 1.5  A_iso_/g_n_β_n_ = -230±2 kG  Γ = 0.5 ± 0.1 mm/s | Central Doublet  S = 0  δ = 0.45±0.01mm/s  ΔE_Q_ = 1.05±0.05 mm/s  Γ = 0.48±0.14 mm/s | NHHS Fe^II^ Doublet  S = 2  δ = 1.4 mm/s  ΔE_Q_ = 3.04±0.05 mm/s  Γ = 0.58±0.01 mm/s | HS Fe^II^ Heme Doublet  S = 2  δ = 0.83±0.06 mm/s  ΔE_Q_ = 2.38±0.05 mm/s  Γ = 0.30 mm/s | Fe^III^ Oxyhydroxide Nanoparticles  S = 5/2 (Superparamagnetic)  δ = 0.51±0.01 mm/s  ΔE_Q_ = 0.55±0.01 mm/s  Γ = 0.42±0.02 mm/s  Or  BPS-Fe Complex  (Initial Relative Area Subtracted)  δ = 0.25 mm/s  ΔE_Q_ = 0.30 mm/s  Γ = 0.30 mm/s  Or  S = 0 [Fe_2_S_2_]^2+^ doublet  δ = 0.29 mm/s  ΔE_Q_ = 0.7 mm/s  Γ = 0.3 mm/s |
| 1 | 0.65 | 0.20 | 0.05 | 0.03 | 0.07 (Fe_2_S_2_]) |
| 2B | 0.16 | 0.07 | 0.72 | 0.05 | 0 |
| 2C | 0.40 | 0.09 | 0.47 | 0.02 | 0.01 (NP) |
| 2D | 0.51 | 0.12 | 0.28 | 0.04 | 0.04 (NP) |
| 2E | 0.54 | 0.13 | 0.26 | 0.02 | 0.04 (NP) |
| 2F | 0.59 | 0.10 | 0.22 | 0.04 | 0.04 (NP) |
| 2G | 0.63 | 0.15 | 0.16 | 0.03 | 0.04 (NP) |
| 4A | 0 | 0 | 0 | 0 | 0 (BPS) |
| 4B | 0 | 0 | 1 | 0 | 0.34 (BPS) |
| 4C | 0 | 0.18 | 0.74 | 0.08 | 0.80 (BPS) |
| 4D | 0 | 0.24 | 0.62 | 0.14 | 0.44 (BPS) |
| 4E | 0.69 | 0.07 | 0.22 | 0.03 | 0.22 (BPS) |
| 4F | 0.67 | 0.06 | 0.23 | 0.04 | 0.29 (BPS) |
| 4G | 0.64 | 0.07 | 0.24 | 0.05 | 0.32 (BPS) |
| 4H | 0.61 | 0.06 | 0.24 | 0.08 | 0.34 (BPS) |
| 4I | 0.66 | 0.08 | 0.20 | 0.06 | 0.31 (BPS) |
| 6A | 0 | 0 | 0 | 0 | - |
| 6B | 0.42 | 0.01 | 0.53 | 0.04 | - |
| 6C | 0.61 | 0.24 | 0.02 | 0.13 | - |
| 6D | 0 | 0 | 0 | 0 | - |
| 6E | <0.01 | 0.01 | 0.65 | 0.33 | - |
| 6F | 0.47 | 0.27 | 0.15 | 0.11 | - |
| 7A | 0 | <0.01 | 0.65 | 0.35 | - |
| 7B | 0 | <0.01 | 0.68 | 0.32 | - |
| 7C | 0.40 | <0.01 | 0.38 | 0.22 | - |
| 7D | 0.40 | <0.01 | 0.32 | 0.20 | 0.08 (NP) |
| 7E | 0.41 | <0.01 | 0.11 | 0.08 | 0.30 (NP) |
| 7F | 0.31 | <0.01 | 0.42 | 0.25 | 0.02 (NP) |
| 7G | 0.65 | 0.16 | 0.15 | 0.04 | - |
| 7H | 0.38 | 0.18 | 0.31 | 0.10 | 0.03 (NP) |
| 7I | 0.62 | 0.25 | 0.10 | 0.03 | - |

**Table S2: Iron concentrations in Experiment 1 samples.** These are n = 1 measurements; we estimate an uncertainty of 25% for each determination.

| Experiment 1  Sample (min) | ^56^Fe  (µM) | ^57^Fe  (µM) | Fe(tot)  (µM) | % ^57^Fe |
| --- | --- | --- | --- | --- |
| 0 | 530 | 11 | 540 | 2 |
| 30 | 450 | 200 | 650 | 31 |
| 60 | 70 | 380 | 450 | 84 |
| 120 | 580 | 440 | 1020 | 43 |
| 180 | 290 | 290 | 580 | 50 |
| 300 | 380 | 360 | 740 | 49 |
| Ave |  |  | 660 ± 140 | 56% (for 60 – 300 min) |

Table S3: Iron concentration of media components.

| Media Component | ^56^Fe (nM) | ^57^Fe (nM) |
| --- | --- | --- |
| Yeast-Nitrogen-Base w/o ammonium sulfate, copper sulfate, or ferric chloride (MP Biomedicals) | 78 ± 2 | 8 ± 2 |
| Ammonium sulfate (Fisher Chemical) | 25 ± 4 | 2 ± 2 |

**Figure S1: Simulation plots** of changes in component concentrations as an iron-deficient yeast cell converts into an iron-replete cell after adding 40 µM [IRON] to the growth media.


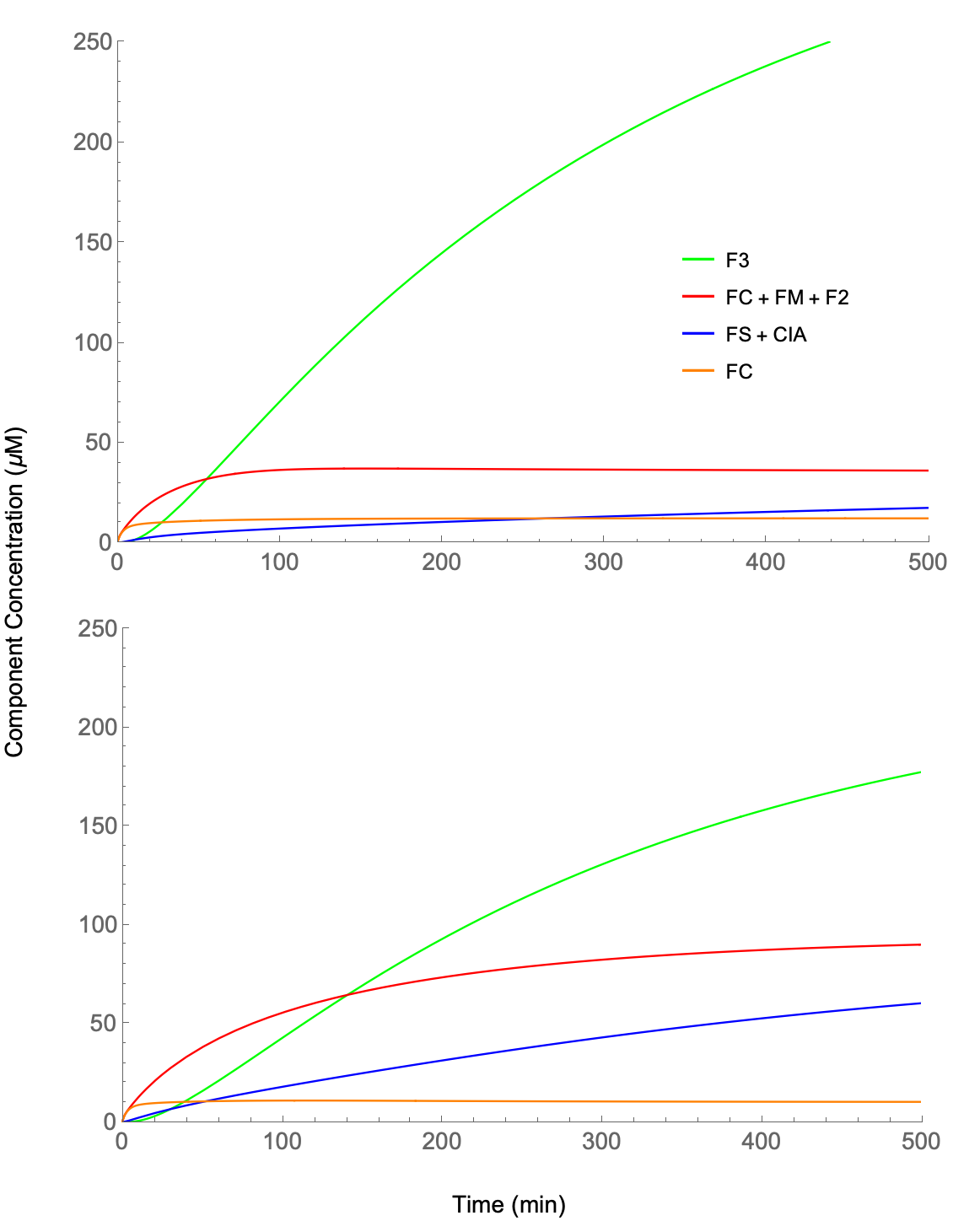


**Figure S2: Growth rate for different media;** no effect of 50% Yeast-Nitrogen-Base or ammonium sulfate in the media on the growth rate.


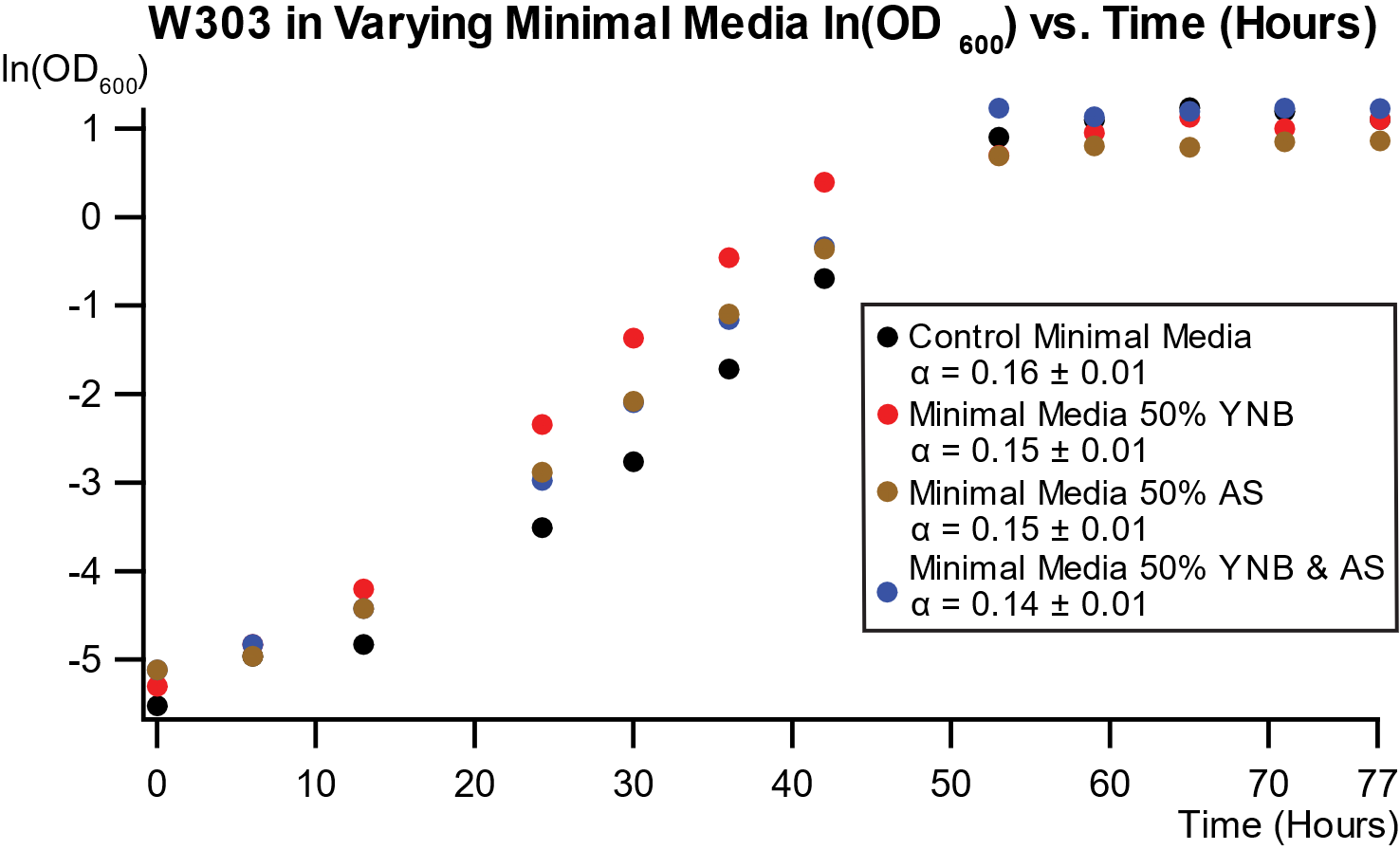

Supplement: Supporting information [file mmc1.docx]
